# The development of suicide risk in people with severe mental disorders during the first year of the COVID-19 pandemic: a claims-based cohort study

**DOI:** 10.1007/s00127-023-02584-z

**Published:** 2023-11-23

**Authors:** Alexander Engels, Janine Stein, Steffi G. Riedel-Heller, Claudia Konnopka, Hans-Helmut König

**Affiliations:** 1 Department of Health Economics and Health Services Research, Center for Psychosocial Medicine, University Medical Center Hamburg-Eppendorf, Martinistr. 52, Building W37, 20246 Hamburg, Germany; 2 Institute for Social Medicine, Occupational Medicine and Public Health, University Medical Center Leipzig, Leipzig, Germany

**Keywords:** Germany, Suicide, COVID-19 pandemic, Administrative claims

## Abstract

**Purpose:**

In this study, we assess how the first and second waves of the COVID-19 pandemic influenced the suicide risk of patients with severe mental disorders in Germany.

**Methods:**

We analyzed German health insurance claims data to compare the suicide risk of patients with severe mental disorders before and during the pandemic. We included *n* = 690,845 patients between October 2019 and March 2020 and *n* = 693,457 patients the corresponding period of the previous year and applied entropy balancing to adjust for confounding covariates. Given that the cause of death was unknown, we defined potential suicides as deaths of patients with a history of intentional self-harm whose passing could not be explained by COVID-19. Potential suicides were tracked in both cohorts over one year and compared using logistic regression.

**Results:**

128 potential suicides were identified in the period during and 101 before the pandemic. This corresponded to a significant increase in the risk for potential suicide of 27.4% compared to the control period (*β* = 0.24, *z* = 1.82, *p* < 0.05).

**Conclusion:**

The noticeable increase in the risk for potential suicide for patients with severe pre-existing mental disorders emphasizes the call for additional efforts to prevent suicide and to help patients cope with their mental illness in the aftermath of the COVID-19 crisis.

**Supplementary Information:**

The online version contains supplementary material available at 10.1007/s00127-023-02584-z.

## Introduction

To reduce the spread of COVID-19, the government in Germany—like in many other countries—imposed numerous restrictions on social contacts. The first lockdown from March 22 until May 5, 2020 encompassed far-reaching measures (e.g., social distancing, contact restrictions, school closures, closure of retail and service companies), which were mostly reimposed on December 16, 2020 until May 2021. It quickly became apparent that patients with pre-existing mental disorders would be particular vulnerable during these prolonged periods of reduced social contacts, self-isolation, and economic losses [1, 2]. While the general population mostly proved to be resilient to the effects of the pandemic (Prati and Mancini, 2021), the mental health of a large proportion of patients with pre-existing conditions worsened considerably [3–7].

One reason for this heterogenous response to social distancing and isolation could be the interpersonal differences in the appraisal of the situation. Multiple studies support the assumption that the negative impact of community mitigation strategies is mediated by the cognitive appraisal (e.g., feeling in control or overwhelmed) and the emotional evaluation of the actions taken (e.g., feeling lonely due to the restrictions) [1, 8, 9]. Furthermore, patients with pre-existing mental disorders rely to a greater extent on formal psychosocial and psychiatric services, which—at least in Germany—were severely restricted during the first months and the subsequent lockdowns of the pandemic [10–12].

The most serious health threat for patients with deteriorating mental health is suicide and self-harm. Patients with severe mental disorders such as depression, bipolar disorder, and schizophrenia have the highest absolute risk of suicide, ranging between 3.7 and 4.9% among women and 5.9 and 7.8% among men within a median follow-up of 18 years [13]. As a result, suicides contribute notably to the elevated mortality of about 10–20 potential life years lost among patients with mental disorders [14, 15]. Given the noticeable increase in suicide attempts [3, 16], suicide ideation and self-harm [17, 18] during the pandemic in several countries, it should be especially important to monitor suicide risk in this vulnerable population. In Germany it is difficult to determine the suicide rate of a particular patient group, because the national mortality database, which documents causes of death, does not contain information on the preceding medical history. Therefore, the German Federal Statistical Office only reports the annual trajectory of the number of suicides in the general population. In addition, little is known about trends in suicide beyond 2020, because the yearly reports of the Statistical Office are published with a considerable time lag. As a result, studies on the influence of the second wave of infections in winter 2020/2021 are lacking, although this period comprised the so far highest recorded death rate due to COVID-19 in Germany and a second restrictive lockdown. Therefore, we used health insurance claims data to compare two large cohorts of patients with severe pre-existing mental disorders before and during the pandemic to determine whether the risk of suicide has increased in the first 12 months of the pandemic.

## Methods

### Study design and data sources

In this retrospective cohort study, we analyzed health insurance claims data for the period from January 1, 2018 to March 31, 2021 from the “Wissenschaftliches Institut der AOK” (“WIdO”). WIdO is the scientific institute of the AOK, which is the largest association of statutory health insurance companies in Germany. In total, the eleven autonomous companies of the AOK cover 26.8 million insurants (reference year 2019). This corresponds to about one third of the German population. To determine the effects of the pandemic, we compared a control cohort treated for a documented diagnosis of severe mental disorder between October 1, 2018 and March 31, 2019 with an exposed pandemic cohort treated for a documented diagnosis of severe mental disorder closely before the pandemic between October 1, 2019 and March 31, 2020. Please note that both cohorts were defined by diagnoses documented within time periods of six months (from October to March) and include indicent and prevalent cases, and that patients with a documented diagnosis within both time intervals could be part of both cohorts. Subsequently, we tracked potential suicides for a 12-month follow-up period starting from April 1, 2019 (control cohort) and April 1, 2020 (pandemic cohort), respectively. The period of 9 months preceding the index diagnoses was used as baseline period in which various covariates were assessed.

### Identification of potential suicides

The outcome of interest was the number of suicides in each cohort. Considering that the cause of death is not documented in claims data, we defined potential suicides as deaths of insurants who had a documented history of intentional self-harm (ICD-10: X60.x – X84.x) between January 1, 2018 and their date of death for the control cohort, or between January 1, 2019 and their date of death for the pandemic cohort. This strategy is based on the empirical evidence that suicide attempts and self-induced injuries occur frequently before fatal suicide attempts and are therefore the best predictor for future suicides [19]. Notably, this strategy does not eliminate the possibility of natural causes of death. Hence, we had to account for factors that could explain excess mortality in the pandemic cohort to obtain unbiased estimates for risk changes. To that end, we employed two different approaches. In our main analysis, we solely excluded deaths of patients who died during a hospital stay due to COVID-19 (ICD-10: U07.1). In addition, we conducted a sensitivity analysis to exclude all deaths due to natural causes by not counting deaths within 30 days after a hospitalization due to anything other than a mental disorder or an injury (ICD-10: Fxx.x, Sxx.x or Txx.x). Nonetheless, we presumably underestimated the actual number of suicides, because not all suicides are preceded by a documented history of self-harm. Primarily, we disregarded suicidal patients who already perished on their first attempt [20].

### Inclusion and exclusion criteria

We restricted the sample to patients with severe mental disorders who were treated for either schizophrenia (ICD-10: F20.x), schizoaffective disorder (F25.x), bipolar disorder (F31.x), severe depression (F32.2, F32.3, F33.2, or F33.3), or personality disorder (F60.x). Given that physicians are legally obligated to encode ‘treatment diagnoses’ for accounting purpose in claims data, documented diagnoses may be less reliable than interview- or survey-based diagnoses [21]. Therefore, we focused on verified claims data diagnoses from university outpatient clinics, dayclinics or hospitals. Patients diagnosed by mental health specialists (i.e., psychiatrists, psychotherapists and neurologists) and other outpatient physicians were only included if the diagnosis was recorded as verified in two consecutive quarters (which is the reason for choosing index periods of six months). We excluded patients who died before the beginning of the follow-up period (*n* = 11,072) and patients with missing information in one of the relevant covariates (*n* = 802).

### Covariates

Even though the cohorts mainly varied in the date of their diagnosis, we decided to control for a large number of covariates to adjust for potential differences at baseline. All covariates were determined for the baseline period of 9 month before the index diagnosis. We controlled for sex, age on the date of the index diagnosis as well as urbanization degree [22] and the federal state of the region of residency. To adress the severity of the disorder and health care utilization, we controlled for the disease groups based on the first established diagnosis during the index period (e.g., schizophrenia, severe depression or bipolar disorder) due to the heterogeneity of the included disorders. In addition, we assessed the number of days at dayclinics or psychiatric clinics, the daily defined doses of antidepressants and antipsychotics, and the number of outpatient visits to psychotherapists and psychiatrists during baseline as well as the number of psychotherapy sessions. Somatic comorbidities were considered by calculating the 21 subscales of the Huber score [23] which is a measure of patients’ chronic disease status based on pharmacy claims data.

### Statistical methods

We employed entropy balancing [24, 25] to reweight the control cohort, so that its covariate moments (i.e., mean, variance, and skewness) mirror the moments of the pandemic cohort in a large range of possible confounding covariates that were determined for the baseline period. If all confounding covariates are considered during entropy balancing, a researcher can simply estimate the causal average treatment effect of an intervention by looking at the difference in the adjusted means. In addition, balancing helps to keep handling of confounders and the modeling process separate, which allows the researcher to report a less complex final model that only includes predictors of interest (e.g., exposure to the pandemic). Subsequently, we applied logistic regression to compare the suicide risk between the control and the pandemic cohort. We tested the one-sided hypothesis that the pandemic increased suicide risk and used weighted maximum likelihood as the estimation technique to incorporate the entropy balancing weights.

## Results

### Sample characteristics

Table 1 depicts the unbalanced sample characteristics for the *n* = 693,457 patients in the control cohort and the *n* = 690,845 patients in the pandemic cohort. The baseline characteristics based on the 9 month prior to the index diagnosis were similar in both cohorts. Approximately 60% of all patients were female and the average age was 55.5 years. Both cohorts consisted of approx. 46% patients with severe depression, 18% with schizophrenia and 18% with a personality disorder. Schizoaffective or bipolar disorders as well as the simultanous diagnosis of multiple severe conditions were observed less frequently. Among those patients who potentially comitted suicide, we found higher rates of severe depression, higher numbers of dispensed antidepressant and antipsychotic prescriptions, and longer inpatient stays when compared to the total sample. Additional characteristics as well as the adjusted sample characteristics after entropy balancing can be found in supplemental tables 1a to 2c.

**Table 1:** Descriptive sample statistics of the most relevant covariates

Category	Outcome	Total sample		Potential suicides	
		Control: *n* = 693,457	Pandemic: *n* = 690,845	Control: *n* = 101	Pandemic: *n* = 128
Socio-demographics	Female	60.24%	60.15%	50.50%	39.06%
	Age	55.52 (17.13)	55.52 (17.06)	59.99 (18.90)	59.99 (20.58)
Region of residency	Major city	27.42%	27.52%	32.67%	28.12%
	Smaller city	36.76%	36.73%	35.64%	33.59%
	Rural area (dense)	18.65%	18.56%	12.87%	15.62%
	Rural area (sparse)	17.17%	17.19%	18.81%	22.66%
Index diagnosis	Bipolar disorder	4.64%	4.70%	6.93%	0.78%
	Multiple diagnoses	9.87%	9.86%	8.91%	11.72%
	Severe depression	45.93%	46.25%	56.44%	54.69%
	Personality disorder	18.36%	18.19%	10.89%	10.16%
	Schizophrenia	17.62%	17.44%	10.89%	18.75%
	Schizoaffective disorder	3.58%	3.56%	5.94%	3.91%
Utilization during baseline	Antidepressants (ddd)	149.08 (230.04)	151.29 (232.11)	211.41 (248.79)	206.85 (282.85)
	Antipsychotics (ddd)	94.78 (235.09)	93.67 (231.06)	134.74 (214.28)	173.69 (396.44)
	Psychiatrist visits	2.56 (6.78)	2.50 (6.49)	2.50 (3.53)	3.16 (4.56)
	Psychotherapy sessions	0.92 (3.96)	0.93 (3.93)	0.37 (2.04)	0.56 (2.84)
	Psychotherapist visits	1.28 (5.15)	1.30 (5.04)	0.58 (2.45)	0.72 (3.41)
	Days in dayclinic	0.93 (7.67)	0.97 (7.85)	1.16 (8.91)	2.37 (13.10)
	Hospital days	4.19 (17.70)	4.37 (18.51)	39.51 (55.10)	28.23 (48.41)

### Deaths during the follow-up period

In Table 2, we report descriptive statistics on potential suicides, COVID-19-related deaths and total deaths in both cohorts. We observed a small increase in the mortality rate from 3.01 to 3.25% in the year of the pandemic. In total numbers, an additional 1,633 patients died in the pandemic cohort of which 632 died in the hospital with a COVID-19 infection. Regarding the number of potential suicides, we report numbers for two separate definitions of potential suicide. In the main analysis (excluding deaths during COVID-19-related inpatient stay), we found that *n* = 101 patients met our criteria for potential suicide in the control cohort compared to *n* = 128 patients in the pandemic cohort. Therefore, the incidence of potential suicides increased from 14.56 per 100,000 patients to 18.53 per 100,000 patients. Interestingly, this increase occurred in men only, and not in patients with bipolar disorder (where the number even decreased from 7 to 1). In the sensitivity analysis (additionally excluding patients with a recent inpatient stay unrelated to mental disorders or injury), we observe *n* = 91 potential suicides in the pandemic and *n* = 61 potential suicides in the control cohort.

**Table 2:** Number, incidence and percentage of potential suicides/deaths related to COVID-19 compared between cohorts

Outcome	Cohort	Potential suicides*		Covid-related deaths	Total deaths
Incidence per 100,000 patients with SMD	Control	14.56	(8.80)	2.60	3006.39
	Pandemic	18.53	(13.17)	91.48	3254.13
Percentage	Control	0.02%	(0.01%)	0.00%	3.01%
	Pandemic	0.02%	(0.01%)	0.09%	3.25%
Number of cases	Control	101	(61)	18	20,848
	Pandemic	128	(91)	632	22,481

*Given that we cannot use claims data to identify suicides with absolute certainty, we report the number of cases for two separate definitions of potential suicide. The main definition counted deaths of patients with a documented history of self-harm whose death was not related to a recent hospital stay due to COVID-19. The second definition further excluded deaths that could be attributable to any hospital stay related to a physical illness. The numbers for the second definition are reported in round brackets; SMD severe mental disorders

**Fig. 1:**
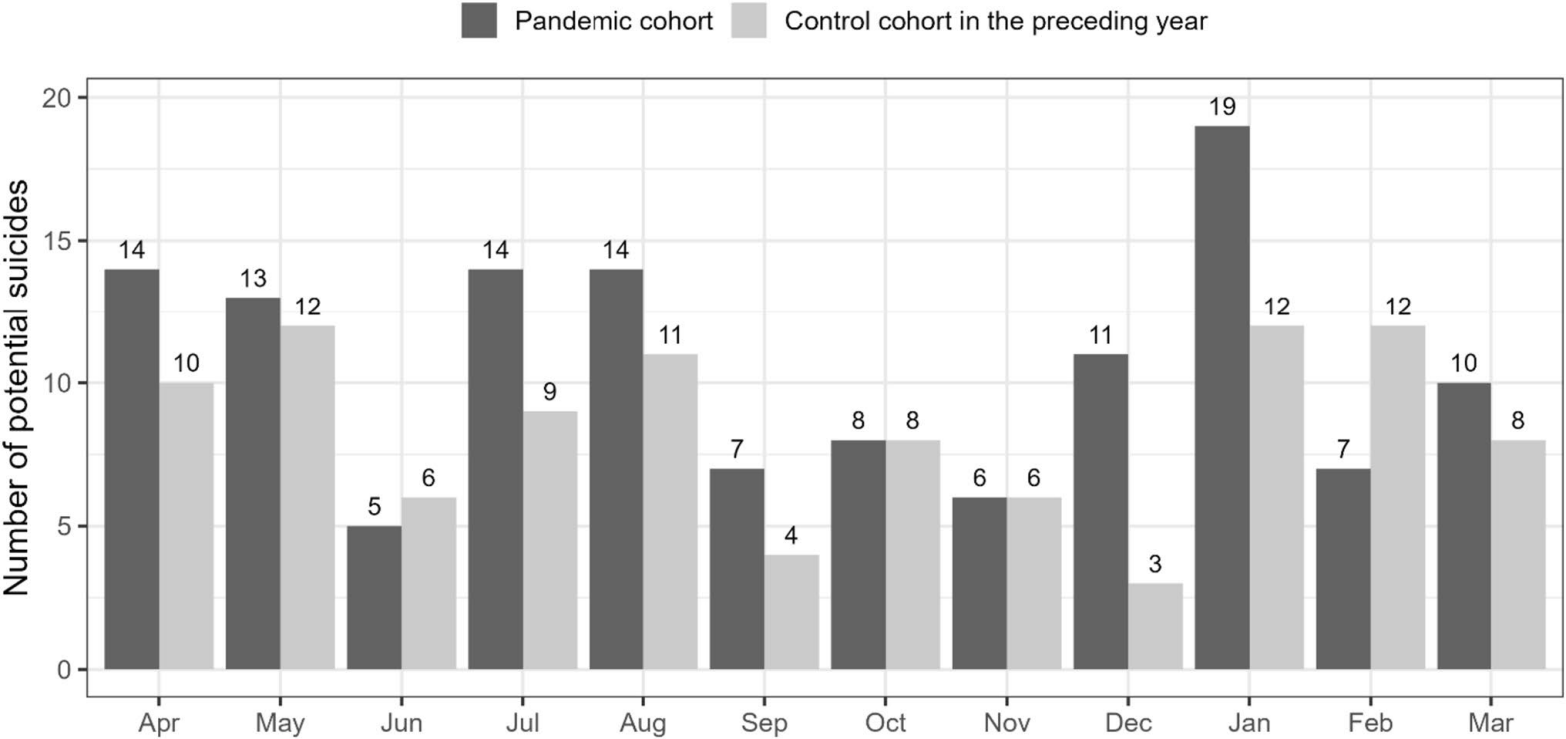
Number of potential suicides in the first year of the pandemic (April 2020 to March 2021) compared to the observed numbers in the year before the pandemic (April 2019–March 2020)

Figure 1 displays the number of potential suicides in each month from April 2020 to March 2021 compared to the respective month of the previous year. The most noticeable differences can be observed in December 2020 and January 2021, which can be characterized by the onset of the second lockdown in Germany starting from the 16th of December.

### Regression analysis

Table 3 presents the findings of the logistic regression. In the main analysis, we found a signficant increase in potential suicide risk of 27.4%, *β* = 0.24, *z* = 1.82, *p* < 0.05. In the sensitivity analysis, we found an even larger increase in potential suicide risk of about 50.3%, *β* = 0.41, *z* = 2.46, *p* < 0.01.

**Table 3:** Logistic regression to determine the association of the pandemic and potential suicide risk

Condition	Term	OR	Estimate	Standard error	*Z*	*p*
Main analysis	(Intercept)		− 8.84	0.10	− 88.56	0.00
	Pandemic cohort	1.27	0.24	0.13	1.82	0.03
Sensitivity analysis	(Intercept)		− 9.34	0.13	− 72.70	0.00
	Pandemic cohort	1.50	0.41	0.17	2.46	0.01

*N* = 1.384.302, OR Odds ratio (reference: control cohort), The main analysis excluded deaths during COVID-19-related inpatient stay whereas the sensitivity analysis excluded all deaths with a recent inpatient stay unrelated to a mental disorder or injury. We rounded all values to 2 decimal places. *p* values refer to a one-sided test

## Discussion

This is the first study that analyzed the association of the COVID-19 pandemic during the first and second lockdown and the risk for suicide in a large cohort of patients with pre-existing severe mental disorders in the whole of Germany. We found an increase of at least 27% in the risk for potential suicide. In addition, our exploratory analysis by calender month suggests a particularly worrying spike in potential suicide risk during the second lockdown period in December 2020 and January 2021.

Given that we could not assess how various risk factors for suicide developed throughout the pandemic, we can only rely on previous studies to try to explain this increase. Undoubtedly, the COVID-19 pandemic affected a range of potential risk factors for suicide [26]. It seems plausible that the economic downturn due to the pandemic increased the risk for deteriorating mental health, alcohol abuse, and suicide [27–29] because more people experienced financial problems, unemployment and worries about the future. This explanation is also supported by a recent study which demonstrated that the deterioration of mental health due to the pandemic could largely be accounted for by occupational and financial changes [30]. This may also be one reason why the increase in the risk for suicide seemed to have occurred mainly in men, for whom financial losses and unemployment may have a stronger impact on mental health in Gemany’s modified male-breadwinner model [31]. In addition, the barriers to access mental healthcare have increased. In the beginning of the pandemic, utilization of psychosocial and psychiatric services declined notably [10, 11]. Dayclinics were closed until appropriate hygiene and safety measures were implemented; and in the outpatient sector, patients often canceled doctor appointments, check-ups and preventive consultations to avoid the risk of an infection [32, 33]. During the longer course of the pandemic, the mental health of many persons gradually decreased, which led to an increased demand for psychosocial services, longer waiting times and as a result a worsened access to care [34]. Furthermore, the prolonged periods of loneliness [1] and the increase in domestic violence during the imposition of restrictions contributed to the deterioration of mental health [35, 36]. Lastly, it is possible that some patients have lost a family member, other kin or acquaintance due to COVID-19 during the second lockdown, as the January 2021 was characterized by the highest death rate due to COVID-19. We have no explanation for the observed decrease in the number of potential suicides among patients with bipolar disorder—maybe this is partly due to statistical uncertainty caused by the relatively small number of obervations in this diagnostic group.

A recent international rapid scoping review on the relation between COVID-19 and suicide in individuals with previously diagnosed mental disorders suggested that the pandemic may have increased the risk for suicidal behavior, especially in patients with major depressive disorder [37]. However, several studies included in this review provided controversial data. Furthermore, there were only few longitudinal studies, and most studies relied on retrospective self-report assessments of changes in suicidal behavior but did not measure the number of actual suicides. A study from Denmark found no significant changes in the hospital-registered rate of suicidal behavior events during the pandemic compared to the pre-pandemic period in people with pre-existing mental disorders [38]. However, the authors pointed out that the registration of suicidal behavior might have been of varying completeness, and that their data did not enable distinction between completed suicides and attempted suicides.

Prior studies conducted in the German general population found no indication of a systematic increase in the number of suicides due to the pandemic [39, 40]. However, in contrast to our study, these studies were confined to smaller regions, were restricted to the year 2020 and did not focus on vulnerable patients groups.

## Strengths and limitations

This study compared two large, representative cohorts that each consisted of almost 700,000 patients with severe mental disorders. The use of claims data allowed us to control for various potential confounders because German claims data contain comprehensive information on sociodemographics, utilization of health services and medical history of each patient as long as it is relevant for billing and accounting purposes. Given that the cause of death is not transmitted to the statutory health insurance, we were only able to apply a proxy for suicide. However, we conducted thorough analyses to rule out other causes of death. We took the excess mortality due to COVID-19 into account, and additionally eliminated the possibility of almost all natural causes of death as long as they occured in hospital.

However, some limitations are worth noting. Given that we could not identity patients who perish on their first attempt to commit suicide, we may have undererstimated the absolute suicide risk in this vulnerable population. Another potential source of bias is that our definition of a potential suicide required that a previous suicide attempt had been documented. It is conceivable that suicide attempts are not always reported as such, e.g., in cases of drug overdose [41, 42]. These factors could explain why we observed an indicidence rate of 18.53 per 100,000 for persons with severe mental disorders, which is only 1.7 times higher than the incidence rate of the general population in Germany in 2020 with 11.07 per 100,000 [43]. We would expect that an unbiased estimate of the incidence rate should be substantially higher considering that we focused on patients with a drastically increased risk for suicide [13]. In fact, we observed an increase in the number of deaths in the pandemic cohort compared to the control cohort by 247.74 per 100,000 (corresponding to an increase of 8%) of which only 88.88 per 100,000 could be attributed to COVID-19—which is compatible with an underestimation of suicides.

## Future reseach

Most limitations of this study are related to the unclear cause of death. Future studies could overcome these limitations by combining the mortality registry with claims data. At present, there are significant hurdles to overcome in order to link information from the mortality registry, which contains information on the cause of death, and health insurance claims data, which contains information on the medical history of an individual [44, 45]. However, an approach based on the mortality registry could be a much more detailed and reliable option to monitor suicides.

## Conclusions

This study highlights the importance of continous monitoring of suicides and mental health in vulnerable populations as the COVID-19 crisis progresses. Whereas suicides for the general population are in line with the trend in previous years, we observed a noticeable increase in the risk for potential suicide for patients with severe pre-existing mental disorders. These findings support the call for additional efforts to prevent suicide and to help patients cope with their mental illness.

### Supplementary Information

Below is the link to the electronic supplementary material.

Supplementary file1 (DOCX 26 kb)

## Data Availability

The datasets supporting the conclusions of this article are owned by the German statutory health insurance AOK. Since public deposition of the data would breach ethical and legal compliance, data are only available upon formal request from the research institute of the AOK (WIdO). To request the data, please contact the institutional body of the WIdO (wido@wido.bv.aok.de). To fulfill the legal requirements to obtain that kind of data, researchers must obtain a permission for a specific research question from the German Federal (Social) Insurance Office. Additionally, researchers must conclude a contract with the statutory health insurance regarding data access, which can be requested from the “AOK-Bundesverband GbR” (Federal Association of Local Health Insurance Funds) under http://aok-bv.de/kontakt/. The licensee is permitted to use the data for the purpose of the research proposal within their company, exclusively. Thereby, a company is defined as an economical unit. Licensees are not allowed to pass the data to a third party, or to create Software or databases with the exception of scientific publications. Moreover, the study has to be approved by the data protection officer both at the statutory health insurance and the research institute.
