# Supplementary material for: The development of suicide risk in people with severe mental disorders during the first year of the COVID-19 pandemic: a claims-based cohort study
Source: Soc Psychiatry Psychiatr Epidemiol. 2023 Nov 23;59(7):1193–200. doi: 10.1007/s00127-023-02584-z (PMC11178617; doi:10.1007/s00127-023-02584-z)
Supplement: Supplementary file 1 — Supplementary file1 (DOCX 26 kb) [file 127_2023_2584_MOESM1_ESM.docx]

| Table 1a: Descriptive sample statistics of the region of residency and the source of the diagnosis | | | | | |
| --- | --- | --- | --- | --- | --- |
|  | | **Total sample** | | **Potential suicides** | |
| **Category** | **Outcome** | **Control: n=693,457** | **Pandemic: n=690,845** | **Control:  n=101** | **Pandemic:  n=128** |
| Socio- demo- graphics | Baden-Württemberg | 15.53% | 15.49% | 13.86% | 15.62% |
|  | Bavaria | 17.16% | 17.29% | 8.91% | 13.28% |
|  | Berlin | 3.38% | 3.24% | 4.95% | 3.91% |
|  | Brandenburg | 2.51% | 2.44% | 1.98% | 2.34% |
|  | Bremen | 0.95% | 0.99% | 1.98% | 2.34% |
|  | Hamburg | 1.69% | 1.67% | 0.99% | 0.78% |
|  | Hesse | 5.88% | 5.92% | 6.93% | 5.47% |
|  | Mecklenburg Western Pomerania | 1.87% | 1.82% | 3.96% | 1.56% |
|  | Lower Saxony | 9.95% | 9.99% | 4.95% | 5.47% |
|  | Northrhine-Westphalia | 20.44% | 20.66% | 19.80% | 14.06% |
|  | Rhineland Palatinate | 4.43% | 4.30% | 6.93% | 7.03% |
|  | Saarland | 0.91% | 0.91% | 2.97% | 2.34% |
|  | Saxony | 6.12% | 6.13% | 8.91% | 7.81% |
|  | Saxony-Anhalt | 2.64% | 2.64% | 3.96% | 4.69% |
|  | Schleswig Holstein | 2.89% | 2.91% | 0.99% | 3.12% |
|  | Thuringia | 3.63% | 3.60% | 7.92% | 10.16% |
| Source of diagnosis | Psychiatric clinic | 6.08% | 5.88% | 46.53% | 43.75% |
|  | Dayclinic | 0.84% | 0.80% | 0.00% | 0.00% |
|  | University outpatient clinic | 0.15% | 0.15% | 0.00% | 0.78% |
|  | Outpatient provider | 42.14% | 42.25% | 22.77% | 24.22% |
|  | Psychiatric outpatient clinic | 14.38% | 14.70% | 10.89% | 12.50% |
|  | Mental health specialist | 36.41% | 36.23% | 19.80% | 18.75% |
| All numbers were rounded to two decimal places. We report the percentage of each value for categorical variables and the mean (standard deviation) for all interval-scaled variables. | | | | | |

| Table 1b: Descriptive sample statistics of the Huber scale | | | | | | | |
| --- | --- | --- | --- | --- | --- | --- | --- |
|  | | **Total sample** | | | **Potential suicides** | | |
| **Category** | **Outcome** | **Control: n=693,457** | **Pandemic: n=690,845** | | **Control:  n=101** | | **Pandemic:  n=128** |
| Huber scale | Acid related disorders | 47.67% | 46.56% | | 61.39% | | 60.94% |
|  | Bone diseases  (osteoporosis) | 1.79% | 1.78% | | 1.98% | | 4.69% |
|  | Cancer | 1.89% | 1.85% | | 6.93% | | 0.78% |
|  | Cardiovascular  diseases (incl.  hypertension) | 63.21% | 63.11% | | 71.29% | | 75.00% |
|  | Dementia | 1.53% | 1.45% | | 1.98% | | 3.12% |
|  | Diabetes mellitus | 14.22% | 14.43% | | 13.86% | | 18.75% |
|  | Epilepsy | 14.70% | 14.80% | | 29.70% | | 25.00% |
|  | Glaucoma | 3.22% | 3.21% | | 5.94% | | 3.12% |
|  | Gout, Hyperuricemia | 4.67% | 4.58% | | 6.93% | | 4.69% |
|  | HIV | 0.22% | 0.24% | | 0.00% | | 0.00% |
|  | Hyperlipidemia | 17.69% | 18.30% | | 15.84% | | 21.09% |
|  | Intestinal inflammatory diseases | 0.76% | 0.77% | | 0.99% | | 0.00% |
|  | Iron deficiency anemia | 2.71% | 2.75% | | 3.96% | | 7.81% |
|  | Migraines | 1.44% | 1.56% | | 0.99% | | 0.00% |
|  | Pain | 27.31% | 27.76% | | 26.73% | | 42.19% |
|  | Parkinson’s disease | 23.05% | 22.27% | | 39.60% | | 49.22% |
|  | Psycholgical disorders  (sleep disorder,  depression) | 48.75% | 49.13% | | 70.30% | | 57.81% |
|  | Psychoses | 40.70% | 40.88% | | 64.36% | | 67.19% |
|  | Respiratory illness  (asthma, COPD) | 13.64% | 13.83% | | 20.79% | | 15.62% |
|  | Rheumatologic  conditions | 29.93% | 29.75% | | 25.74% | | 28.91% |
|  | Thyroid disorders | 26.57% | 26.88% | | 32.67% | | 31.25% |
|  | Tuberculosis | 0.05% | 0.06% | | 0.00% | | 0.00% |
| All numbers were rounded to two decimal places. We report the percentage of each value for categorical variables and the mean (standard deviation) for all interval-scaled variables. | | | | | | | |
| Table 2a: Descriptive sample statistics of the most relevant control variables after the adjustment through entropy balancing | | | | | | | |
|  | | **Cohort type: Control** | | | | **Pandemic** | |
| **Category** | **Outcome** | **Control: n=693,457** | | **Adj. Control:  n=693,457** | | **Adj. Pandemic:  n=690,845** | |
| Socio- demo- graphics | female | 60.24% | | 60.15% | | 60.15% | |
|  | age | 55.52 (17.13) | | 55.64 (17.06) | | 55.64 (17.06) | |
| Region of residency | Major city | 27.42% | | 27.52% | | 27.52% | |
|  | Smaller city | 36.76% | | 36.73% | | 36.73% | |
|  | Rural area (dense) | 18.65% | | 18.56% | | 18.56% | |
|  | Rural area (sparse) | 17.17% | | 17.19% | | 17.19% | |
| Diagnosis at baseline | Bipolar disorder | 4.64% | | 4.70% | | 4.70% | |
|  | Multiple diagnoses | 9.87% | | 9.86% | | 9.86% | |
|  | Severe depression | 45.93% | | 46.25% | | 46.25% | |
|  | Personality disorder | 18.36% | | 18.19% | | 18.19% | |
|  | Schizophrenia | 17.62% | | 17.44% | | 17.44% | |
|  | Schizoaffective disorder | 3.58% | | 3.56% | | 3.56% | |
| Utilization during the preperiod | Antidepressants (ddd) | 149.08 (230.04) | | 151.29 (232.11) | | 151.29 (232.11) | |
|  | Antipsychotics (ddd) | 94.78 (235.09) | | 93.67 (231.07) | | 93.67 (231.06) | |
|  | Psychiatrist visits | 2.56 (6.78) | | 2.50 (6.49) | | 2.50 (6.49) | |
|  | Psychotherapy sessions | 0.92 (3.96) | | 0.93 (3.93) | | 0.93 (3.93) | |
|  | Psychotherapist visits | 1.28 (5.15) | | 1.30 (5.04) | | 1.30 (5.04) | |
|  | Days in dayclinic | 0.93 (7.67) | | 0.97 (7.85) | | 0.97 (7.85) | |
|  | Hospital days | 4.19 (17.70) | | 4.37 (18.51) | | 4.37 (18.51) | |
| All numbers were rounded to two decimal places. We report the percentage of each value for categorical variables and the mean (standard deviation) for all interval-scaled variables. | | | | | | | |

| Table 2b: Descriptive sample statistics of the region of residency and the source of the diagnosis after the adjustment through entropy balancing | | | | |
| --- | --- | --- | --- | --- |
|  | | **Cohort type: Control** | | **Pandemic** |
| **Category** | **Outcome** | **Control: n=693,457** | **Adj. Control:  n=693,457** | **Adj. Pandemic:  n=690,845** |
| Socio- demo- graphics | Baden-Württemberg | 15.53% | 15.49% | 15.49% |
|  | Bavaria | 17.16% | 17.29% | 17.29% |
|  | Berlin | 3.38% | 3.24% | 3.24% |
|  | Brandenburg | 2.51% | 2.44% | 2.44% |
|  | Bremen | 0.95% | 0.99% | 0.99% |
|  | Hamburg | 1.69% | 1.67% | 1.67% |
|  | Hesse | 5.88% | 5.92% | 5.92% |
|  | Mecklenburg Western Pomerania | 1.87% | 1.82% | 1.82% |
|  | Lower Saxony | 9.95% | 9.99% | 9.99% |
|  | Northrhine-Westphalia | 20.44% | 20.66% | 20.66% |
|  | Rhineland Palatinate | 4.43% | 4.30% | 4.30% |
|  | Saarland | 0.91% | 0.91% | 0.91% |
|  | Saxony | 6.12% | 6.13% | 6.13% |
|  | Saxony-Anhalt | 2.64% | 2.64% | 2.64% |
|  | Schleswig Holstein | 2.89% | 2.91% | 2.91% |
|  | Thuringia | 3.63% | 3.60% | 3.60% |
| Source of diagnosis | Psychiatric clinic | 6.08% | 5.88% | 5.88% |
|  | Dayclinic | 0.84% | 0.80% | 0.80% |
|  | University outpatient clinic | 0.15% | 0.15% | 0.15% |
|  | Outpatient provider | 42.14% | 42.25% | 42.25% |
|  | Psychiatric outpatient clinic | 14.38% | 14.69% | 14.70% |
|  | Mental health specialist | 36.41% | 36.23% | 36.23% |
| All numbers were rounded to two decimal places. We report the percentage of each value for categorical variables and the mean (standard deviation) for all interval-scaled variables. | | | | |

| Table 2c: Descriptive statistics of the huber scale after the adjustment through entropy balancing | | | | |
| --- | --- | --- | --- | --- |
|  | | **Cohort type: Control** | | **Pandemic** |
| **Category** | **Outcome** | **Control: n=693,457** | **Adj. Control:  n=693,457** | **Adj. Pandemic:  n=690,845** |
| Huber scale | Acid related disorders | 47.67% | 46.56% | 46.56% |
|  | Bone diseases  (osteoporosis) | 1.79% | 1.78% | 1.78% |
|  | Cancer | 1.89% | 1.85% | 1.85% |
|  | Cardiovascular  diseases (incl.  hypertension) | 63.21% | 63.11% | 63.11% |
|  | Dementia | 1.53% | 1.45% | 1.45% |
|  | Diabetes mellitus | 14.22% | 14.43% | 14.43% |
|  | Epilepsy | 14.70% | 14.80% | 14.80% |
|  | Glaucoma | 3.22% | 3.21% | 3.21% |
|  | Gout, Hyperuricemia | 4.67% | 4.58% | 4.58% |
|  | HIV | 0.22% | 0.24% | 0.24% |
|  | Hyperlipidemia | 17.69% | 18.30% | 18.30% |
|  | Intestinal inflammatory diseases | 0.76% | 0.77% | 0.77% |
|  | Iron deficiency anemia | 2.71% | 2.75% | 2.75% |
|  | Migraines | 1.44% | 1.56% | 1.56% |
|  | Pain | 27.31% | 27.76% | 27.76% |
|  | Parkinson’s disease | 23.05% | 22.27% | 22.27% |
|  | Psycholgical disorders  (sleep disorder,  depression) | 48.75% | 49.13% | 49.13% |
|  | Psychoses | 40.70% | 40.88% | 40.88% |
|  | Respiratory illness  (asthma, COPD) | 13.64% | 13.83% | 13.83% |
|  | Rheumatologic  conditions | 29.93% | 29.76% | 29.75% |
|  | Thyroid disorders | 26.57% | 26.87% | 26.88% |
|  | Tuberculosis | 0.05% | 0.06% | 0.06% |

| All numbers were rounded to two decimal places. We report the percentage of each value for categorical variables and the mean (standard deviation) for all interval-scaled variables. |
| --- |
